# Magnolol and Honokiol Inhibited the Function and Expression of BCRP with Mechanism Exploration

**DOI:** 10.3390/molecules26237390

**Published:** 2021-12-06

**Authors:** Chung-Ping Yu, Pei-Ying Li, Szu-Yu Chen, Shiuan-Pey Lin, Yu-Chi Hou

**Affiliations:** 1 School of Pharmacy, College of Pharmacy, China Medical University, Taichung 406040, Taiwan; yu1095813@gmail.com (C.-P.Y.); anag8066@gmail.com (P.-Y.L.); joywork0920@gmail.com (S.-Y.C.); 2 Department of Pharmacy, China Medical University Hospital, Taichung 404332, Taiwan; 3 College of Medical and Health Science, Asia University, Taichung 41354, Taiwan

**Keywords:** magnolol, honokiol, BCRP, EGFR, MDR

## Abstract

Breast cancer resistance protein (BCRP), one of the ATP-binding cassette (ABC) transporters, was associated with the multidrug resistance (MDR) of chemotherapy. Magnolol (MN) and honokiol (HK) are major bioactive polyphenols of *Magnolia officinalis*. This study investigated the effects of MN and HK on the function and expression of BCRP for the purpose of developing BCRP inhibitor to overcome MDR. Cell lines including MDCKII-BCRP and MDCKII-WT were used for evaluating the function and expression of BCRP. The results showed that MN (100–12.5 µM) and HK (100–12.5 µM) significantly decreased the function of BCRP by 80~12% and 67~14%, respectively. In addition, MN and HK were verified as substrates of BCRP. Furthermore, MN and HK reduced the protein expression of BCRP, and inhibited the phosphorylation of epidermal growth factor receptor (EGFR) and phosphatidylinositol 3-kinase (PI3K). In conclusion, both MN and HK decreased the function and expression of BCRP via EGFR/PI3K signaling pathway. Therefore, both compounds were promising candidates for reversing the MDR of chemotherapy.

## 1. Introduction

In recent decades, it is well recognized that overexpression of ATP-binding cassette (ABC) transporters, such as P-glycoprotein (P-gp), multidrug resistance-associated proteins (MRPs) and breast cancer resistance protein (BCRP), is one of the causative mechanisms of multidrug resistance (MDR) [1]. The inhibition on ABC transporter-mediated efflux from cancer cells was believed to be one of the feasible strategies to overcome MDR [2]. Many phytochemicals such as flavonoids, curcuminoids, taccalonolides, and terpenes have been reported to show inhibitions on ABC transporters. Therefore, there are growing interests to search for natural products as inhibitors of P-gp, MRPs and BCRP [3,4,5].

BCRP is the most recently reported ABC transporter and its tissue distribution highly resembles P-gp in tumors and various organs, and shared broad specificity of substrates [6]. The substrates of BCRP included a variety of therapeutic agents such as anticancer drugs (mitoxantrone, irinotecan, methotrexate, gefitinib), antiviral drugs (lamivudine, zidovudine), antihyperlipidemic drugs (rosuvastatin), antidiabetic drugs (glyburide) and anti-inflammatory drugs (sulfasalazine) as well as many acidic physiological substances such as estrone-3-sulfate, 17β-estradiol 17-(β-D-glucuronide), and uric acid [7]. Thus, BCRP has now been recognized by the USFDA to be one of the key ABC transporters involved in clinically relevant drug disposition, drug interactions [7] and might also play important roles in MDR [8].

**Figure 1:**
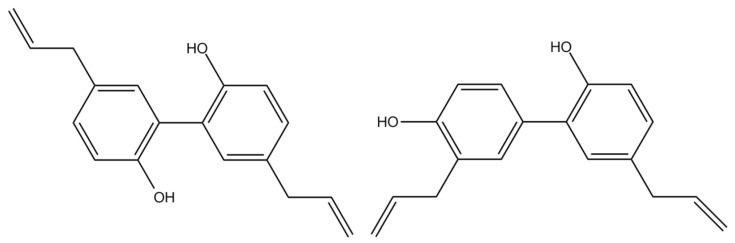
Chemical structures of MN (**left**) and HK (**right**).

A variety of natural compounds have been investigated in the efforts of developing MDR-reversing agents owing to their low toxicities [9]. Magnolol (MN) and honokiol (HK) (chemical structures shown in Figure 1), major bioactive polyphenols of *Magnolia officinalis*, have been shown to be safe and without troublesome adverse effects [10]. In pharmacological aspect, MN exhibited numerous beneficial pharmacological activities, such as anti-inflammatory [11,12], neuroprotection [13], antitumor [14,15], cardiovascular protection [16], anti-angiogenesis [17], antioxidation [18] and antibacterial activities [19]. Being a structural isomer of MN, HK also showed various beneficial biological effects, including neuroprotection [20], antitumor [21], anti-angiogenesis [22], antioxidation and antibacterial activities [23]. Concerning the associations with MDR, MN and HK were promising agents for reducing MDR via down-regulation of P-gp expression [24]. Moreover, MN and HK were inhibitors of nuclear factor-kappa B (NF-κB) and reversed the MDR of vinblastine, a substrate of P-gp [25].

It has been recognized that epidermal growth factor receptor (EGFR) was overexpressed during the progression of a number of tumor types [26], and thus, suppressing the EGFR signaling pathways was an effective therapeutic target in treating cancers [27,28]. On the other hand, the induction of BCRP expression by EGF treatment has been demonstrated in several cell lines, including human BCRP-transfected Madin-Darby canine kidney cells (MDCKII-BCRP) [9,10]. Therefore, we speculated that BCRP expression was associated with EGFR/PI3K signaling pathway. Till now, the modulations of MN and HK on BCRP have not been reported yet. This study investigated the influences of MN and HK on the function and expression of BCRP. Furthermore, the modulation mechanisms were explored.

## 2. Results

### 2.1. Cell Viability Assay

MTT assay indicated that various concentrations of tested drugs and Ko143, a potent BCRP inhibitor used as positive control, at 0.25 μM all exerted no toxic influences on the viability of MDCKII-WT and MDCKII-BCRP cells (Supplementary Materials).

### 2.2. Effects of MN and HK on the Function of BCRP

**Figure 2:**
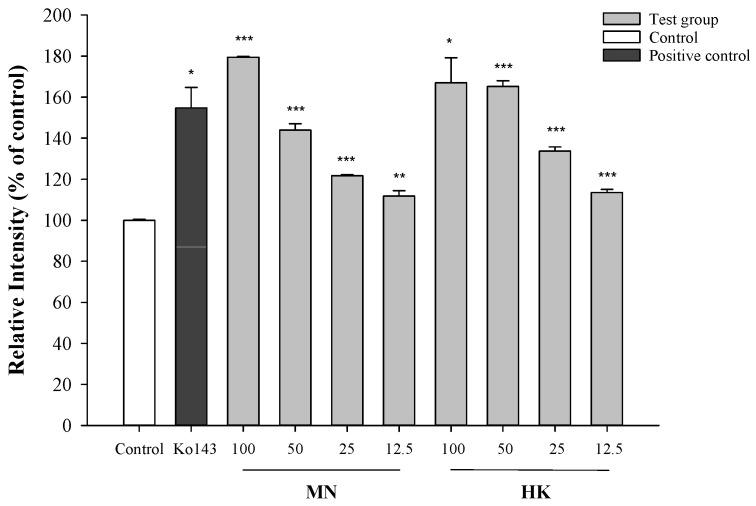
Effects of MN (μM), HK (μM) and Ko143 (0.25 μM, a positive control of BCRP inhibitor) on the intracellular accumulations of MXR in MDCKII-BCRP cells by using flow cytometry method. * *p* < 0.05, ** *p* < 0.01, *** *p* < 0.001. Control: 0.01% DMSO in reaction buffer.

The effects of MN and HK on the intracellular accumulation of mitoxantrone (MXR), a typical BCRP substrate, in MDCKII-BCRP cells by using flow cytometry method are shown in Figure 2. The results showed that MN and HK at 100, 50, 25 and 12.5 μM significantly increased the intracellular accumulations of MXR by 80%, 44%, 22%, 12% (MN), and 67%, 65%, 34%, 14% (HK), respectively. Ko143 at 0.25 μM significantly increased the intracellular accumulations of MXR by 55%.

### 2.3. Intracellular Accumulations of MN and HK in MDCKII-WT and MDCKII-BCRP Cells

**Figure 3:**
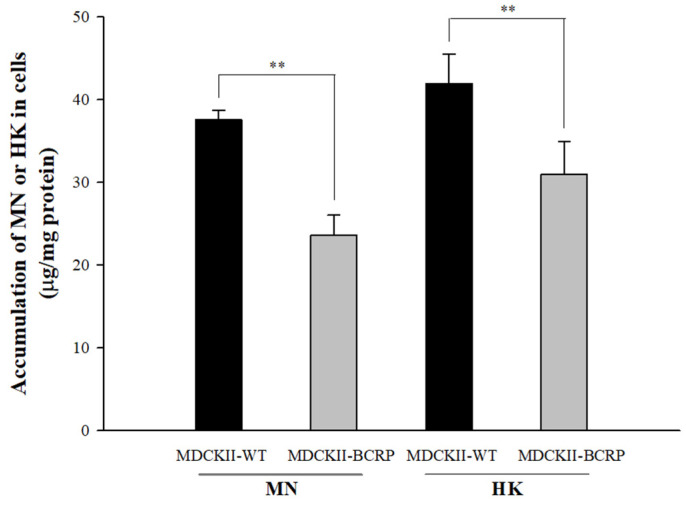
Intracellular accumulations of MN (100 μM) and HK (100 μM) in MDCKII-WT and MDCKII-BCRP cells after incubation for 30 min determined by HPLC-UV analysis and corrected with protein contents. ** *p* < 0.01.

An HPLC-UV analytical method was established and validated in this study for the determination of MN and HK in cell lysates. The calibration ranges of MN (2.5–80 μg/mL) and HK (1.3–40 μg/mL) in cell lysates were with good linearity (the correlation coefficients of MN and HK were 0.9998 and 0.9996, respectively). The intracellular accumulations of MN and HK in MDCKII-WT and MDCKII-BCRP cells after incubations with MN and HK are shown in Figure 3. The intracellular concentrations of MN in MDCKII-BCRP cells after 30-min incubations were significantly lower than the corresponding concentrations in MDCKII-WT cells by 38%. Likewise, the intracellular concentration of HK in MDCKII-BCRP cells was significantly lower than the corresponding concentrations in MDCKII-WT cells by 27%. The results indicated that MN and HK are substrates of BCRP.

### 2.4. Effects of MN and HK on the Expressions of BCRP, EGFR, and PI3K

**Figure 4:**
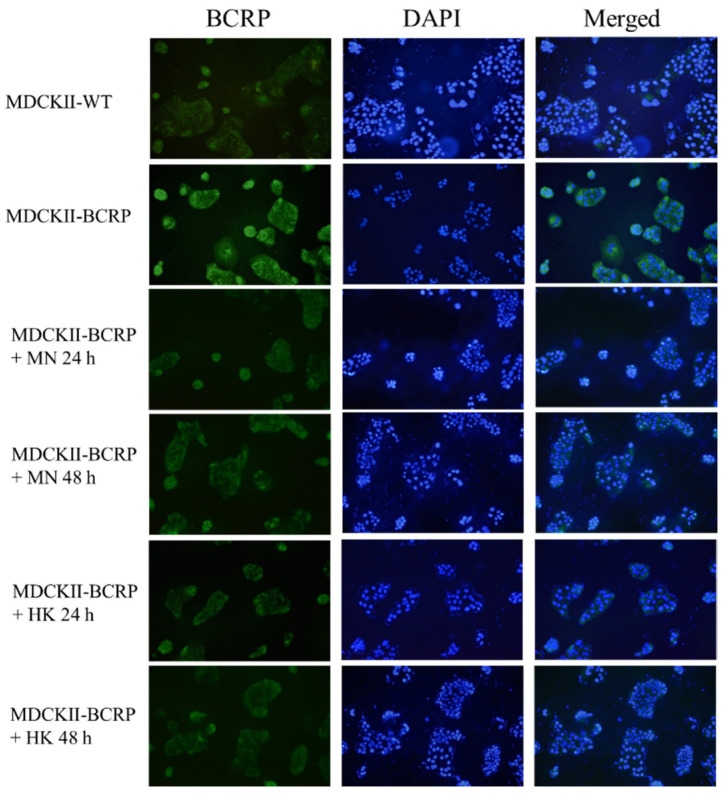
Effects of MN (12.5 μM) and HK (12.5 μM) on the protein expression of breast cancer resistance protein (BCRP) in MDCKII-WT and MDCKII-BCRP cells at 24 h and 48 h after incubation.

By using immunofluorescence assay, the expressions of BCRP in MDCKII-WT and MDCKII-BCRP cells after treating with MN and HK, individually, for 24 h and 48 h, are shown in Figure 4. The results indicated that MN and HK decreased the expression of BCRP in MDCKII-BCRP cells.

**Figure 5:**
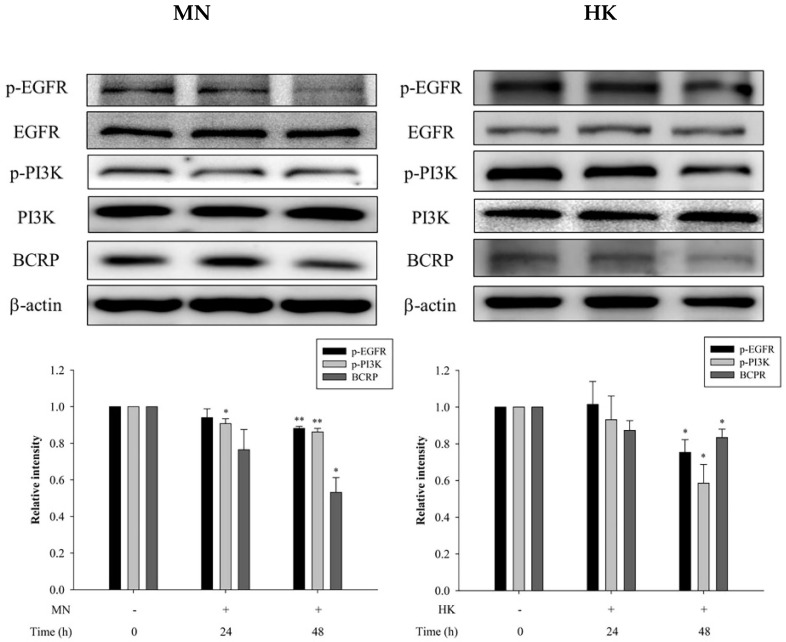
Effects of MN (12.5 μM) and HK (12.5 μM) on the protein expressions of p-EGFR, EGFR, p-PI3K, PI3K and BCRP, in MDCKII-BCRP cells at 24 h and 48 h after incubation. * p < 0.05, ** p < 0.01.

On other hand, through western blot analysis, the protein expressions of p-EGFR, p-PI3K and BCRP in MDCKII-BCRP cells after treating with MN and HK, individually, for 24 h and 48 h are shown in Figure 5. The results showed that after treatment with MN and HK for 48 h, the protein expressions of p-EGFR were significantly reduced by 12% and 25%, the protein expressions of p-PI3K were significantly decreased by 14% and 41%, and the protein expressions of BCRP were significantly reduced by 47% and 17%, respectively, indicaing that MN and HK decreased the expressions of p-EGFR, p-PI3K and BCRP. Moreover, Figure 5 also showed that the modulation effects were in a time-dependent manner.

## 3. Discussion

This study employed cell model to investigate the involvement of MN and HK in the BCRP-mediated transport and their modulation mechanism on BCRP. MXR was used as a substrate of BCRP. The transport assays showed that both MN and HK increased the intracellular accumulation of MXR, proving that MN and HK acted as inhibitors of BCRP. In order to verify whether MN and HK were substrates of BCRP, the intracellular accumulations of MN or HK in MDCKII-WT and MDCKII-BCRP cells were compared. The results showed that the intracellular accumulations of MN and HK in MDCKII-BCRP cells were significantly lower than those in MDCKII-WT cells, indicating that the efflux transports of MN and HK were mediated by BCRP. Taken together, MN and HK were substrates and inhibitors of BCRP.

In order to understand the modulation mechanism of MN and HK on BCRP, the influences on the EGFR/PI3K signaling pathway cells were investigated by using MDCKII-BCRP cells. The present results showed that MN and HK inhibited the protein expressions of p-EGFR and p-PI3K, thereby inhibiting BCRP expression, indicating that MN and HK down-regulated BCRP via the EGFR/PI3K signaling pathway. Recently, MN and HK have been reported as promising anticarcinogenic and anticancer agents through inhibiting PI3K/AKT/mammalian target of rapamycin (mTOR) signaling pathway [29]. In addition, MN triggered the apoptosis of human prostate cancer cells by suppressing the EGFR/PI3K/AKT signaling pathway [30]. Similarly, HK inhibited proliferation, invasion, and induced apoptosis through EGFR/PI3K/AKT signaling pathway [31]. Besides, MN and HK were shown to inhibit the protein expression of NF-κB, which was one of the major downstream targets of EGFR/PI3K and involved in a variety of processes, such as inflammatory, immune responses and MDR [32,33,34,35]. Therefore, our present results indicating that MN and HK inhibited EGFR/PI3K signaling pathway was in good agreements with these previous findings [29,30,31].

The PI3K/AKT/mTOR pathway was a key link modulating the MDR of cancers [34,36,37]. Moreover, if this signaling pathway was blocked, BCRP expression would be inhibited, and the MDR was probably reversed [27]. The present study revealing that MN and HK decreased the function and expression of BCRP through inhibiting the phosphorylation of EGFR and PI3K suggested that MN and HK were promising candidates for overcoming the MDR of chemotherapy using BCRP substrate drugs. In brief, this was the first study to demonstrate that MN and HK were substrates/inhibitors of BCRP and worthy of further investigations as a single anticancer agent or in combined therapeutics with other anticancer drugs such as mitoxantrone, topotecan and gefitinib etc. [38,39].

## 4. Materials and Methods

### 4.1. Chemicals and Reagents

MN (purity > 98%) and HK (purity > 98%) were purchased from ChemFaces (Wuhan, PRC). Ko143 (purity 96%) was obtained from Enzo Life Sciences, Inc. (Farmingdale, NY, USA). MXR (purity 99%) was obtained from Santa Cruz Biotechnology, Inc. (Santa Cruz, CA, USA). Dimethyl sulfoxide (DMSO), formic acid, sodium dodecyl sulfate (SDS), 3-(4′,5′-dimethylthiazol-2′-yl)-2,5-diphenyltetrazolium bromide (MTT), butylparaben and triton X-100 were supplied by Sigma-Aldrich Chemical Co. (St. Louis, MO, USA). Fetal bovine serum (FBS) was obtained from Biological Industries Inc. (Kibbutz, Beit Haemek, Israel). Penicillin-Streptomycin-Glutamine, Dulbecco’s modified Eagle medium (DMEM), 4-(2-hydroxyethyl)-1-piperazineethanesulfonic acid, Hank’s buffered salt solution (HBSS)and trypsin/EDTA were purchased from Invitrogen (Grand Island, NY, USA). Antibodies against ABCG2 and GAPDH were purchased from GeneTex (San Antonio, TX, USA). Polyvinylidene fluoride transfer membranes (Immobilon P) and chemiluminescence (ECL) were obtained from Millipore Corp. (Bedford, MA, USA). 4′,6-diamidino-2-phenylindole (DAPI) staining solution and goat anti-rabbit IgG H&L (DyLight™ 488) antibody were purchased from Abcam (Cambridge, UK). Acetonitrile (ACN) and methanol with LC grade were obtained from Mallinckrodt Baker (Phillipsburg, NJ, USA). Milli-Q plus water (Bedford, MA, USA) was used throughout this study.

### 4.2. Cell Lines and Culture Conditions

MDCKII-WT and MDCKII-BCRP cells were kindly provided by Prof. Dr. Piet Borst (Netherlands Cancer Institute, Amsterdam, Netherlands). Cells were grown in Dulbecco’s modified Eagle medium supplemented with 10% fetal bovine serum, 100 units/mL of penicillin, 100 μg/mL of streptomycin and 292 μg/mL of glutamine at 37 °C in a humidified incubator containing 5% CO_2_. The medium was changed every other day and cells were subcultured when 80% to 90% confluency was reached.

### 4.3. Cell Viability Assay

The effects of MN, HK and Ko143 on the viability of MDCKII-WT and MDCKII-BCRP cells were evaluated by MTT assay, modified from a previous study [40]. After seeding the cells into a 96-well plate for overnight incubation, these tested drugs were added and incubated for suitable time depending on each experimental design, then 100 μL of MTT (5 mg/mL) was added and incubated for additional 3 h. DMSO solution was added to lyze the cell and then the cell viability was measured at 595 nm by a microplate reader (BioTek instruments lnc., Winooski, VT, USA).

### 4.4. Effects of MN and HK on the Function of BCRP

MDCKII-BCRP cells were used to evaluate the effects of MN and HK on the efflux transport of MXR, a fluorescent typical substrate of BCRP [41]. Briefly, cell suspension (5 × 10^5^ in each reaction tube) was pre-incubated with a series concentration of MN and HK, and Ko143 (0.25 μM, an inhibitor of BCRP) at 37 °C for 15 min. MXR (5 μM) was then added and co-incubated for another 30 min. After incubation, cells were washed and re-suspended in ice-cold phosphate buffered saline. The intracellular fluorescence of MXR was measured by a FACScan flow cytometer. The transport studies were performed in triplicates.

### 4.5. The Intracellular Accumulations of MN and HK in MDCKII-WT and MDCKII-BCRP Cells

In order to verify whether MN and HK are substrates of BCRP, MDCKII-WT and MDCKII-BCRP cells were used for comparison. The cells were seeded onto 12-well plates at a density of 3 × 10^5^ cells per well. After 3-day culturing, the medium was removed and washed with ice-cold phosphate-buffered saline. Four hundred microliters of MN or HK (100 μM in pH 7.4 HBSS) was added into each well and incubated at 37 °C for 30 min. After washing, cell lysates were obtained after trypsinization with 300 μL of 0.5% Trypsin-EDTA and lyzed by liquid nitrogen. The cell lysate (100 μL) was added to 100 μL of pH 5.0 buffer, 50 μL of ascorbic acid, 50 μL of 0.1 N HCl and partitioned with 300 μL of ethyl acetate containing 40 μg/mL of butylparaben as internal standard. After centrifugation, the ethyl acetate layer was dried by nitrogen gas and reconstituted with 50 μL of acetonitrile, and 20 μL was subject to HPLC-UV analysis. For calibrator preparation, cell lysate (100 μL) was spiked with various concentrations of MN (2.5–80.0 μg/mL) or HK (1.3–40.0 μg/mL) and then mixed with 100 μL of pH 5.0 buffer, 50 μL of ascorbic acid, 50 μL of 0.1 N HCl, then partitioned with 300 μL of ethyl acetate containing 40 μg/mL of butylparaben. The later procedure was identical to that described above for cell lysates. The intracellular concentrations of MN and HK were calculated after correction with protein contents.

### 4.6. Effects of MN and HK on the Expression of BCRP

Cells were seeded into 12-wells plates at a density of 1 × 104/wells and incubated with MN (12.5 μM) and HK (12.5 μM), individually, at 37 °C for 24 h and 48 h [42]. After the supernatant was removed, methanol was added and stood for 10 min. After washing, 0.1% Triton X-100 was incubated for 10 min, washed 3 times with PBS, and incubated with 1% bovine serum albumin (BSA) for 1 h. Cells were then washed 3 times in PBS, and then incubated with primary antibody overnight at 4 °C. After washing, cells were incubated with the DyLight™ 488-conjugated goat anti-rabbit IgG antibody (Jackson ImmunoResearch, West Grove, PA, USA) at room temperature for 2 h. After washing, cells were incubated with the 4′,6-diamidino-2-phenylindole (DAPI) for 10 min in the dark. The imaging of cells was acquired by using confocal laser scanning microscopy (Nikon, TE2000-U, Tokyo, Japan).

### 4.7. Effects of MN and HK on BCRP Expression and EGFR/PI3K Signaling Pathway

After treatment with MN (12.5 μM) and HK (12.5 μM) for 24 h and 48 h, individually, the cells were lyzed with radioimmune precipitation (RIPA) buffer (Merck, Darmstadt, Germany) and collected. The samples were separated by 10% SDS-polyacrylamide gel electrophoresis and then transfered onto polyvinylidenedifluoride membranes (Immobilon, Millipore, Bedford, MA, USA). The membranes were blocked at room temperature for 1 min in the blocking buffer (Goal Bio, Taipei, TW), and then washed 3 times with 0.1% TBST (Tris-buffered saline with 0.1% Tween^®^ 20 detergent). After washing, the blots were incubated with p-EGFR, EGFR, p-PI3K, PI3K, BCRP and β-actin primary antibodies, individually, at 4 °C overnight. Then the blots were washed 3 times with 0.1% TBST, and reacted with secondary antibodies at room temperature for 1 h. The bands were detected by using the ECL kit (Advansta Inc., San Jose, CA, USA).

### 4.8. Data Analysis

The statistical software SPSS was used for analyzing the differences among treatments by using unpaired Student’s *t*-test, taking *p* < 0.05 as significant.

## 5. Conclusions

MN and HK decreased the function and expression of BCRP through inhibition on the EGFR/PI3K signaling pathway. This is the first study revealing that MN and HK were substrates/inhibitors of BCRP and they are potential candidates to overcome MDR of BCRP substrate drugs.

## Data Availability

The data that support the findings of this study are not available.

## Supplementary Materials

The following are available online. Figure S1: Effects of MN (μM) and HK (μM) on the cell viability of MDCKII-WT and MDCKII-BCRP at 1 h. (Mean ± S.D.) Control: 0.01% DMSO in reaction buffer, Figure S2: Effects of MN (μM) and HK (μM) on the cell viability of MDCKII-BCRP at 24 and 48 h. (Mean ± S.D.) Control: 0.01% DMSO in reaction buffer.

## Sample Availability

Samples of the compounds in this study are available from the authors.

## Abbreviations

ABC, ATP-binding cassette; ACN, acetonitrile; AKT, protein kinase B; BCRP, breast cancer resistance protein; DAPI, 4′,6-diamidino-2-phenylindole; DMEM, Dulbecco’s modified Eagle medium; DMSO, dimethyl sulfoxide; ECL, chemiluminescence; EGF, epidermal growth factor; EGFR, EGF receptor; ERK, extracellular signal regulated kinase; FBS, fetal bovine serum; HBSS, Hank’s buffered salt solution; HK, honokiol; MAPK, mitogen-activated protein kinase; MDCKII, Madin-Darby canine kidney type II cells; MDR, multidrug resistance; MEK, mitogen-activated protein kinase; MN, magnolol; MRPs, multidrug resistance-associated proteins; mTOR, mammalian target of rapamycin; MTT, 3-(4′,5′-dimethylthiazol-2′-yl)-2,5-diphenyltetrazolium bromide; MXR, mitoxantrone; NF-κB, nuclear factor-kappa B; P-gp, P-glycoprotein; PI3K, phosphatidylinositol 3-kinase; SDS, sodium dodecyl sulfate; TKIs, tyrosine kinase inhibitors.
