# Supplementary material for: Magnolol and Honokiol Inhibited the Function and Expression of BCRP with Mechanism Exploration
Source: Molecules. 2021 Dec 6;26(23):7390. doi: 10.3390/molecules26237390 (PMC8659015; doi:10.3390/molecules26237390)
Supplement: Supplementary file 1 [file molecules-26-07390-s001.zip › molecules-1467209-supplementary.pdf]

Supplementary Materials

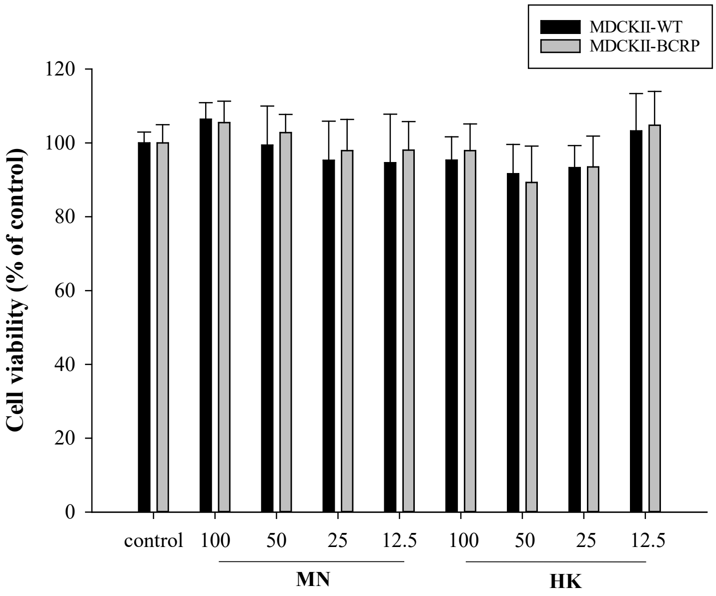

Figure S1. Effects of MN (μM) and HK (μM) on the cell viability of MDCKII-WT and MDCKII-BCRP at 1 hr. (Mean ± S.D.) Control: 0.01% DMSO in reaction buffer.

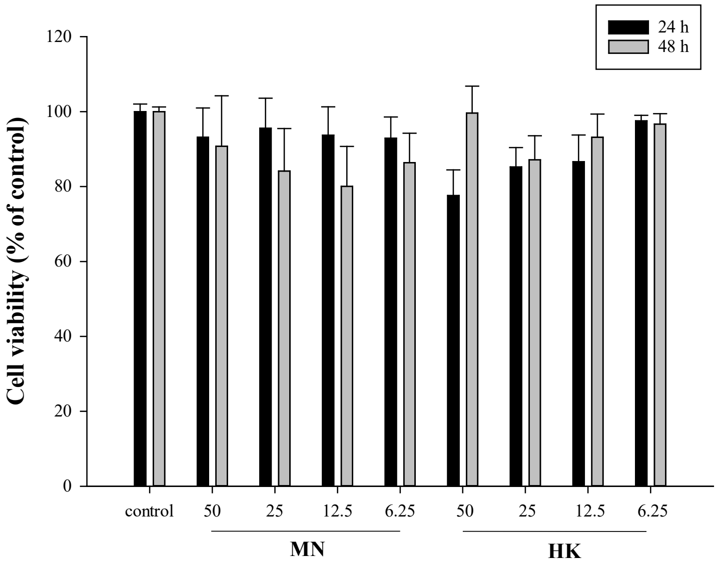

Figure S2. Effects of MN (μM) and HK (μM) on the cell viability of MDCKII-BCRP at 24 and 48 hr. (Mean ± S.D.) Control: 0.01% DMSO in reaction buffer.
